# Long-term follow-up of early cleft maxillary distraction

**DOI:** 10.1186/s40902-016-0069-x

**Published:** 2016-05-03

**Authors:** Young-Wook Park, Kwang-Jun Kwon, Min-Keun Kim

**Affiliations:** Department of Oral and Maxillofacial Surgery, College of Dentistry, Gangneung-Wonju National University, 7 Jukheon-gil, Gangneung, 210-702 South Korea

**Keywords:** Unilateral complete cleft lip and palate, Maxillary constriction, Early distraction treatment

## Abstract

**Background:**

Most of cleft lip and palate patients have the esthetic and functional problems of midfacial deficiencies due to innate developmental tendency and scar tissues from repeated operations. In these cases, maxillary protraction is required for the harmonious facial esthetics and functional occlusion.

**Case presentation:**

A 7-year old boy had been diagnosed as severe maxillary constriction due to unilateral complete cleft lip and palate. The author tried to correct the secondary deformity by early distraction osteogenesis with the aim of avoiding marked psychological impact from peers of elementary school. From 1999 to 2006, repeated treatments, which consisted of Le Fort I osteotomy and face mask distraction, and complementary maxillary protraction using miniplates were performed including orthodontics. But, final facial profile was not satisfactory, which needs compromising surgery.

**Conclusions:**

The result of this study suggests that if early distraction treatment is performed before facial skeletal growth is completed, an orthognathic surgery or additional distraction may be needed later. Maxillofacial plastic and reconstructive surgeons should notify this point when they plan early distraction treatment for cleft maxillary deformity.

## Background

The midfacial hypoplasia or maxillary constriction is a common secondary deformity in congenital cleft deformity involving primary palate. The causes of the midfacial hypoplasia or maxillary constriction are innate growth impairment [1] and scar contracture engaged in hard palate during the palate repair [2]. Despite of orthodontic treatment, up to 25 % of patients with cleft lip and palate needs surgical interventions to achieve balanced and harmonious facial appearance [3].

Traditional approach to manage the cleft maxillary deformity is orthognathic surgery, which sometimes has difficulties to achieve the surgical goal due to the skeletal clefting and excessive soft tissue scarring. Moreover, Le Fort I advancement and miniplate fixation in adult patients with cleft lip and palate deformity showed a mean skeletal relapse of 23 % even though autogenous iliac bone graft had been performed [4].

After the pioneering study [5, 6], the maxillary distraction technique is considered as the valuable alternative to orthognathic surgery for patients with maxillary constriction secondary to orofacial cleft [7, 8]. Moreover, this technique can be applicable during the period of mixed dentition, which is appealing for whom the wait for the skeletal maturity could be psychologically unendurable.

Now, the present author reports a long-term clinical result of early maxillary distraction, i.e., distraction during mixed dentition for a patient with unilateral cleft lip and palate. The rationale of the early distraction was not only psychological relieve of the patient but also with the purpose of guiding normal maxillomandibular relation until skeletal maturation. The aim of this study was to analyze the affecting factors for successful outcome in cleft maxillary distraction treatment and to provide a particular clinical experience which might influence surgeon’s choice of treatment strategy: conventional osteotomy versus distraction osteogenesis.

## Case presentation

**Fig. 1:**
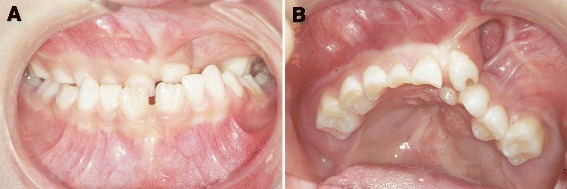
Pretreatment intraoral photograms at the time of initial diagnosis presenting severe maxillary constriction and anterior crossbite (**a**) and the nasolabial fistula (**b**) taken on Feb 25, 1999

The patient was born in 1992 with a complete unilateral cleft lip and palate and first visited to the Department of Oral and Maxillofacial Surgery, Gangneung-Wonju National University Dental Hospital in 1997. He had undergone cheiloplasty 5 months after birth and palatoplasty at the age of 17 months in other hospital. He had no accompanying anomalies and also no other specific medical history. We diagnosed him as a severe maxillary constriction with anterior crossbite and intraoral nasolabial fistula (Fig. 1) and planned maxillary distraction for early correction of patient’s maxillomandibular relation.

**Fig. 2:**
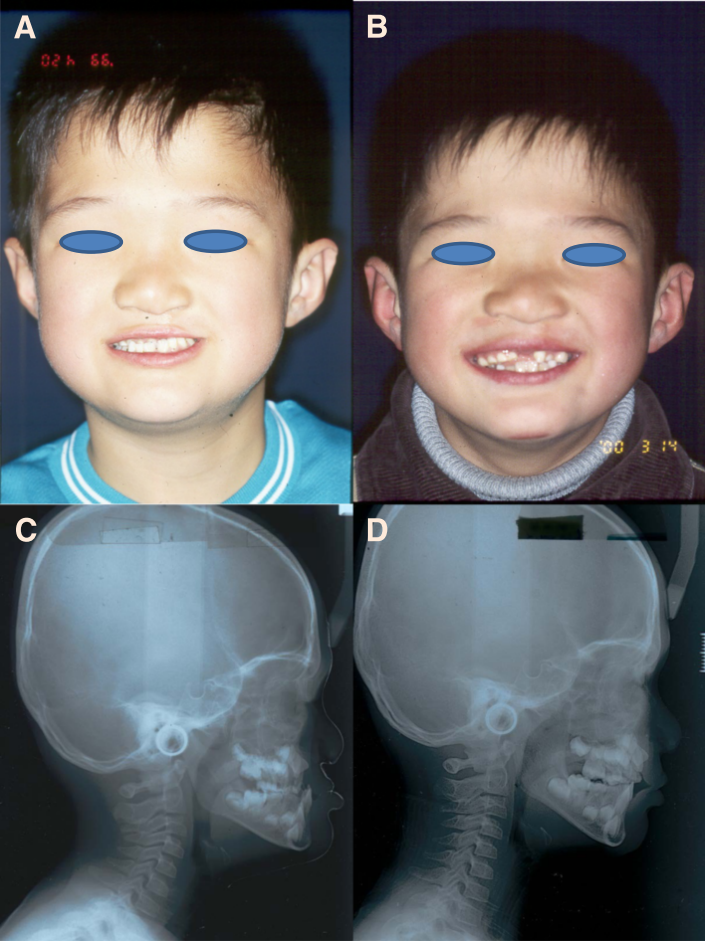
Pre-distraction and post-distraction frontal facial views (**a**, **b**) and lateral cephalograms (**c**, **d**). On Jul 28, 1999, a high-level maxillary Le Fort I osteotomy with separation of pterygomaxillary junction was performed. Seven days after the osteotomy, face mask distraction was performed with external elastic force of 1000 g per side for 1 month to achieve the desired maxillary position and occlusion. The direction of the force was almost parallel to the occlusal plane

On Jul 28, 1999, a high-level transverse maxillary Le Fort I osteotomy just below the infraorbital foramen was performed to avoid injuring the unerupted permanent tooth buds. The pterygomaxillary junction was separated, but maxillary down-fracturing was not performed. No movement of the osteotomized segments or internal fixation was achieved intraoperatively. The halo portion of the distraction device was placed after the closure of the surgical wound. Seven days after the operation, face mask distraction was applied with external elastic force of 1000 g per side for 1 month to achieve the desired maxillary position. The amount of maxillary advancement was 10 mm at A point. The direction of maxillary protraction was almost parallel to the palatal plane. After 6-month retention period, we finished the early distraction treatment (Fig. 2). We concomitantly delivered a chin cup for the purpose of restriction of mandibular growth with full-time orthopedic force of 400 g per side. After maxillary protraction, we applied a fan type active plate for maxillary expansion for 11 months for occlusal interdigitation.

**Fig. 3:**
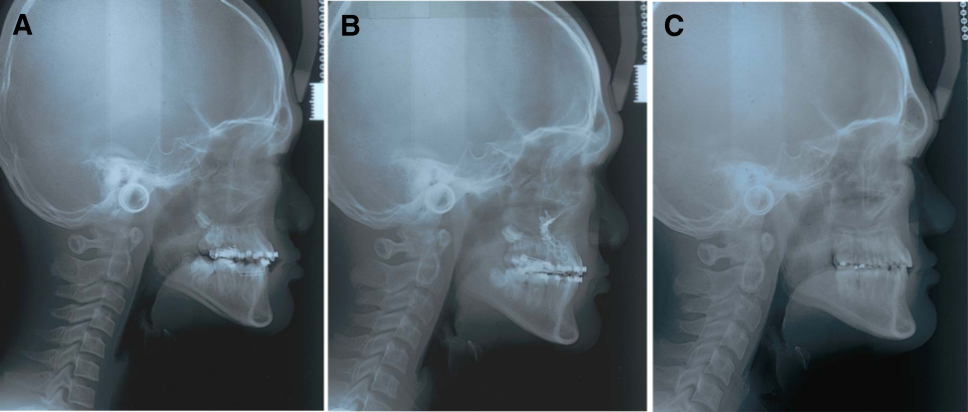
We performed complementary maxillary protraction using miniplates as the skeletal anchorage. On May 18, 2005, seven-holed curved miniplates were fixed to the zygomatic buttress. Three weeks after the operation, the orthopedic force of 400 g per side for 6 months was applied using a protraction head gear. The direction of the force was 30° downward to the occlusal plane. Pre-protraction (**a**), after miniplate fixation (**b**), and post-protraction (**c**) lateral cephalograms

On Apr 17, 2002, alveolar bone grafting was performed as the canine was erupting using cancellous iliac crestal bone. As the mandible was growing, the position of the maxilla was getting deficient for ideal maxillomandibular position. So, complementary protraction of the maxilla was planned using miniplate as a skeletal anchorage. On May 18, 2005, seven-holed curved miniplates (M4 Rigid Fixation System, OsteoMed, USA) were fixed to the thick zygomatic buttress area with three 6-mm screws under general anesthesia. The lower end of the plate was exposed to the oral cavity via attached gingiva between maxillary canine and the first premolar. Three weeks after the operation, the orthopedic force of 400 g per side for 6 months was applied at least longer than 12 h per day with the use of protaction head gear. The direction of the orthopedic force was 30° downward to the occlusal plane (Fig. 3).

**Fig. 4:**
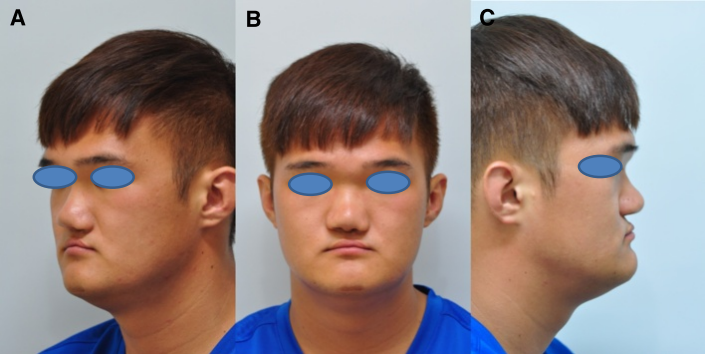
Post-treatment (maxillary distraction and complementary protraction) oblique (**a**), frontal (**b**), and profile (**c**) views taking on Jul 21, 2014

**Fig. 5:**
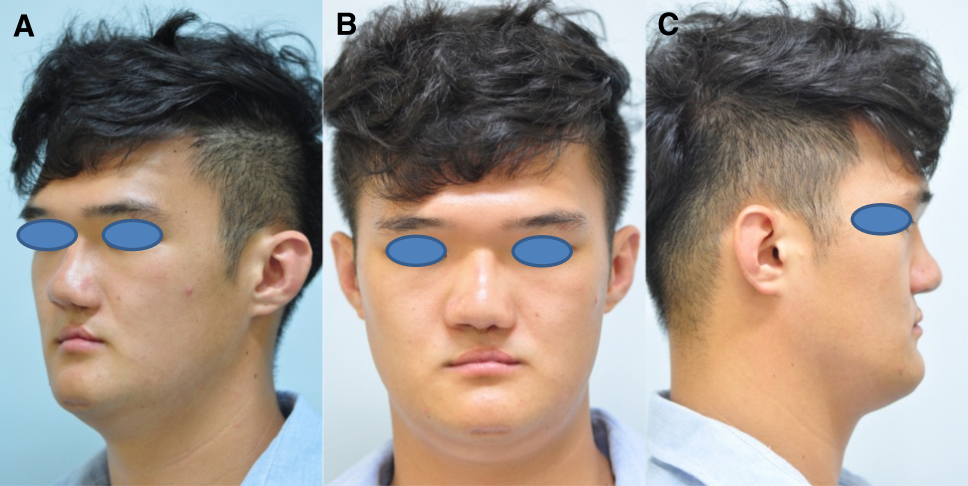
On Jan 9, 2015, the patient underwent reduction genioplasty, paranasal augmentation, and corrective rhinoplasty using autogenous rib cartilage. Oblique (**a**), frontal (**b**), and profile (**c**) views 8 months after compromising contouring surgery

After protraction treatment, the mandible showed an anterior and superior rotation with loss of anterior facial height, and upward inclination of the occlusal plane was detected (Table 1). Facial profile was not satisfactory due to the hyperplastic mandible and prominent frontal bossing (Fig. 4). To compromise patient’s profile, facial contouring surgery was planned. On Jan 9, 2015, reduction genioplasty, paranasal augmentation, and corrective rhinoplasty were performed, and 8-month follow-up photograms are presented in Fig. 5.

**Table 1:** Cephalometric measurements after maxillary distraction and protraction treatment

Measurement	Unit	Norm^a^	2011-01-12
SNA	degree	82.48	75.3
SNB	degree	80.42	85.1
ANB	degree	2.05	−9.8
Angle of convexity	degree	2.36	−26.5
Mandibular length	mm	121.8	133.8
Midfacial Length	mm	93.6	89.2
Mandibular plane	degree	22.75	14.0
Occlusal plane-SN	degree	15.24	0.9
Palatal plane angle	degree	0.5	−10.4
Gonial angle	degree	130.0	121.2
Lower anterior facial height	mm	76.34	75.3
Nasolabial angle	degree	105.0	70.2
Y-axis to FH	degree	61.72	51

^a^Normal measurements of Korean people

(The council of the faculty of orthodontics. Textbook of orthodontics, 2nd edi Seoul: Daehannarae; 2006. p.186–187.)

### Discussion

Management of severe cleft maxillary constriction presents a challenge for maxillofacial plastic and reconstructive surgeons. Age, status of maxillary segments, amount of required maxillary protraction, type of distraction device, vector control, and stability should be carefully considered when surgeons plan cleft maxillary distraction treatments. In this presenting case, the overall result was not satisfactory for ideal patient’s profile, and the causes are discussed.

Cheung et al. concluded distraction osteogenesis tends to be preferred to conventional osteotomy for younger cleft lip and palate patients with severe maxillary deformities in a clinical study [9]. In cleft patients with maxillary deformity, distraction osteogenesis was commonly performed in their age of 6 to 15 [10]. At initial diagnosis and treatment plan, our hypothesis was that early established normal occlusion would guide normal maxillomandibular relation at the end stage of maxillofacial growth. But, in this study, established normal occlusal interdigitation during mixed dentition had not maintained during the period of mandibular growth spurt. Also, it was not clear to decide the amount of maxillary protraction considering individual growth potential. As a result, patient profile was not improved, which needs compromising contouring surgery. Practically, it was not persuasive to restart preoperative orthodontic treatment for orthognathic surgery after completing distraction treatment. So, we had chosen the compromising surgery and finalize the tedious treatment.

Cleft maxillary distraction would be more effective if the alveolar bone grafting was performed beforehand [11]. We performed the distraction treatment before alveolar bone grafting. So, we connected the alveolar segments by resin splint before applying the distraction force. Nonetheless, distraction force seemed to push the segments to the alveolar gap, thereby decreasing the amount of maxillary protraction. Also, we had used a face mask to transfer the distraction force because the more effective RED (external regid fixation) system [12, 13] had not been so popular that time especially to children at school age. In this present case, face mask distraction which used the teeth as a support, showed limited effect for ideal and suitable three-dimensional movement of the maxillary segment.

After face mask distraction, as the mandible was growing, we needed more maxillary space for ideal occlusion and maxillomandibular relation. So, we pioneerly applied miniplate as a skeletal anchorage for maxillary protraction [14, 15]. Seven-holed curved miniplates successfully transferred the protraction force to the maxilla. But, face mask protraction lacked exact vector control and finally dentoalveolar compensation developed. Also, protraction face mask and miniplate anchorage seemed to be weak to overcome the tensile force from palatal scar in this particular case.

## Conclusions

In summary, the author presents a clinical outcome of repeated treatments for secondary maxillary constriction of unilateral cleft lip and palate. In these growing patients, the appropriate degree of correction could not be predicted. And, there was no evidence that corrected occlusion during mixed dentition could guide normal maxillomandibular relation at the end stage of maxillofacial growth. Therefore, the result of this study suggests that if early distraction treatment is performed before facial skeletal growth is completed, an orthognathic surgery or additional distraction may be needed later. Maxillofacial plastic and reconstructive surgeons should notify this point when they plan early distraction treatment for cleft maxillary deformity.

## Consent

Written informed consent was obtained from the patient for publication of this case report and any accompanying images.
